# Loss of Drosophila *UBE3A* phenocopies Piezo dysfunction and drives hyperphagic feeding in Drosophila

**DOI:** 10.1080/19336934.2026.2616950

**Published:** 2026-01-16

**Authors:** Benjamin Geier, Logan Neely, Eli Coronado, Lawrence T. Reiter

**Affiliations:** a Department of Physiology, Tulane University, New Orleans, LA, USA; b Graduate Program in Neuroscience, Tulane University, New Orleans, LA, USA; c MSRF Program, UTHSC, Memphis, TN, USA; d Department of Biology, Christian Brothers University, Memphis, TN, USA; e Tulane Brain Institute, Tulane University, New Orleans, LA, USA

**Keywords:** Angelman syndrome, UBE3A, Piezo, hyperphagia, satiety signaling, ion channel

## Abstract

Angelman syndrome (AS) is a rare neurogenetic disorder characterized by developmental delay, speech impairment, ataxia, epilepsy, and in some cases hyperphagic feeding behavior. AS is caused by loss of function mutations, loss of expression, or maternal allele deletion of the E3 ubiquitin ligase *UBE3A*. Recent work has identified a connection between UBE3A and the mechanosensitive ion channel PIEZO2, raising the possibility that UBE3A may regulate PIEZO-dependent satiety signaling. In this study, we investigated the role of the Drosophila UBE3A ortholog, *Dube3a*, in Piezo-associated feeding behaviors. Single-cell RNA-sequencing data revealed overlapping expression of *Dube3a* and *Piezo* within crop and enterocyte populations of the gut, identifying a relevant cellular context for this pathway to occur. We developed a novel feeding assay using GFP-expressing yeast to quantify food intake and gut distention *in vivo*. *Dube3a* loss-of-function (*Dube3a*^*15b*^) flies exhibited hyperphagia and gut distention nearly identical to *Piezo* knockout flies. Analysis of chromosomal deficiency lines spanning the *Dube3a* locus further supported a requirement for *Dube3a* in normal satiety signaling. Finally, biochemical analyses demonstrated that *Dube3a* knockdown results in decreased Piezo protein levels, consistent with an indirect regulatory relationship. Together, these findings identify *Dube3a* as a critical regulator of Piezo-dependent satiety pathways and suggest that dysregulation of mechanosensory signaling may contribute to hyperphagia observed in AS. Further work is needed to define the intermediate factors linking UBE3A activity to Piezo stability and function.

## Introduction

Angelman syndrome (AS) is a rare neurogenetic disorder with an incidence of 1/15,000 births and characterized by severe developmental delays, speech impairments, ataxic movements, epilepsy, and frequent laughter [1]. AS arises from the loss of expression of a single paternally imprinted copy of the *UBE3A* gene, a HECT domain E3 ubiquitin ligase, also known as E6AP [2,3]. While the majority of AS cases occur as a result of deletion of the maternal 15q11.2-q13.1 locus [4], loss-of-function (LOF) mutations that exclusively affect *UBE3A* on the maternal allele will also result in AS [5]. Biochemical studies investigating point mutations in *UBE3A* reveal that any loss or reduction of catalytic activity plays a critical role in the development of AS [6]. Large maternal deletions comprise 75% of all AS cases, point mutations of maternal *UBE3A* comprise 20% of cases, imprinting centre defects make up 3%, and paternal uniparental disomy (pUPD) causes up to 2% of causes [7]. Previous work suggested that, unlike the more common deletion class, only pUPD patients display hyperphagic feeding behavior [8,9]. However, recent large-scale AS cohort studies suggest this hyperphagic phenotype may be present in all AS individuals [10,11].

A new connection between UBE3A and the mechanosensitive ion channel PIEZO2 has recently been established. In that study, loss of *Ube3a* in an AS mouse model and human AS cell lines resulted in decreased PIEZO2 activity and protein expression [12]. Piezo proteins are pore-forming subunits of ion channels that activate in response to mechanical stimuli [13]. The human genome encodes two channel isoforms, PIEZO1 and PIEZO2, which are expressed in a myriad of tissues (kidneys, vasculature, Merkel cells, and others) [14–18]. In *D. melanogaster* there is only one copy of the Piezo ion channel gene, but it shares equal homology for both mammalian isoforms [19]. As in mammals, Drosophila express *Piezo* in the crop (stomach) and intestine. Knockout of *Piezo* in Drosophila results in a severe gut distention phenotype, indicative of hyperphagia [20,21]. The high homology between fly and mammalian Piezo, in addition to the connection between UBE3A and PIEZO2 recently described in mice, prompted us to look for a connection between *Dube3a* and *Piezo* in flies by capitalizing on the hyperphagia and gut defects associated with AS.

Here, we investigated the role of *Dube3a* (the fly UBE3A homolog) in hyperphagic feeding behavior. We improved on the existing dye-based Drosophila feeding methodologies by introducing a novel feeding assay that incorporates green fluorescent protein (GFP) expressing yeast, allowing for quantitative analysis of gut distention. Using this new assay, we show connections between *Dube3a*, *Piezo* and hyperphagia. Furthermore, to further support the relationship between Dube3a and Piezo, we generated fly lines that express GFP-tagged Piezo while simultaneously altering *Dube3a* expression. These studies provided evidence that UBE3A could regulate PIEZO2 levels providing a new avenue for potential therapeutics of AS-related hyperphagia.

## Results

### In silico *scRNA-seq analysis of Drosophila gut cells confirms co-expression of* Dube3a *and* Piezo

**Figure 1.**
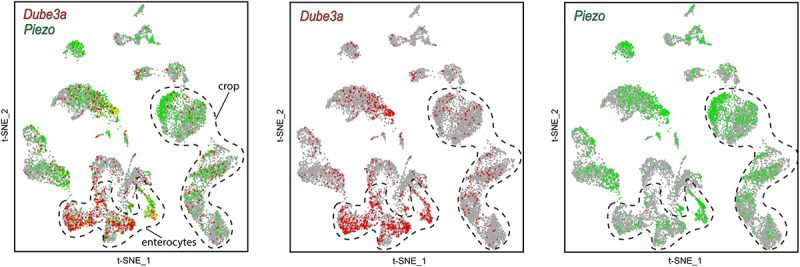
scRNA-seq reveals overlapping expression of *Piezo* and *Dube3a* in Drosophila gut cell populations. *in silico* analysis of Drosophila gut scRNA-seq dataset (visualized using t-distributed stochastic neighbor embedding, t-SNE) shows distinct clustering of annotated gut cell subtypes (dotted outlines). Feature plots indicate *Piezo*-expressing cells (green) and *Dube3a*-expressing cells (red). Overlapping expression of both genes is observed within enterocyte clusters and a crop-associated cell cluster, supporting shared cellular expression contexts in gut tissues. Data accessed via the SCope portal (scope.Aertslab.org).

Although a functional link between Piezo and UBE3A has been described, whether these genes are expressed within the same cells of the digestive system has not been examined. Because *Piezo* is highly expressed throughout the gut and plays a key role in feeding regulation, we leveraged publicly available single-cell RNA-sequencing (scRNA-seq) data from the SCope database (scope.aertslab.org) [22,23], to assess the cellular overlap between *Piezo* and *Dube3a* expression. We analyzed annotated gut cell populations, focusing on enterocytes and crop-associated cells, which are directly implicated in satiety signaling [24–26]. T-distributed stochastic neighbor embedding (t-SNE) visualization revealed robust co-expression of *Piezo* and *Dube3a* transcripts across all analyzed gut clusters (Figure 1). The overlapping expression of these genes within enterocytes and crop cells supports a shared cellular context providing a potential link that may account for the hyperphagia seen in AS patients.

### Dube3a *loss of function flies display gut distention phenotype indicative of hyperphagia*

**Figure 2.**
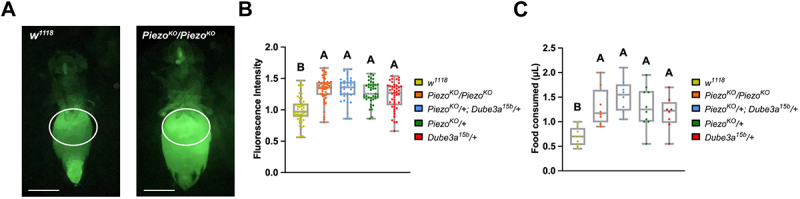
*Dube3a* LOF recapitulates *Piezo*^*KO*^ gut distention phenotype. A) image of *Piezo*^*KO*^ homozygote to *w*^*1118*^ fly after ingesting GFP+ yeast. Region of interest (white outline) for fluorescent normalization and quantification. Scale bar 500 µM. B) feeding analysis of *Piezo*^*KO*^*/+*; *Dube3a*^*15b*^*/+* (*n* = 37); *Piezo*^*KO*^*/Piezo*^*KO*^ (*n* = 48), *Piezo*^*KO*^*/+* (*n* = 41), *Dube3a*^*15b*^*/+* (*n* = 45), and *w*^*1118*^ (*n* = 51). Significant gut distention phenotypes were observed in all groups when compared to *w*^*1118*^ control flies. *Piezo*^*KO*^*/Piezo*^*KO*^ flies did not show significant distention versus *Dube3a*^*15b*^ and *Piezo*^*KO*^*/+* flies. C) CAFE feeding analysis of *Piezo*^*KO*^*/+*; *Dube3a*^*15b*^*/+* (*n* = 8); *Piezo*^*KO*^*/Piezo*^*KO*^ (*n* = 8), *Piezo*^*KO*^*/+* (*n* = 10), *Dube3a*^*15b*^*/+* (*n* = 8), and *w*^*1118*^ (*n* = 9). Significant food consumption was observed in all groups when compared to *w*^*1118*^ control flies. *Piezo*^*KO*^*/+; Dube3a*^*15b*^*/+* flies did not show significant distention versus *Piezo*^*KO*^*/Piezo*^*KO*^ and *Piezo*^*KO*^*/+* flies. Each data point represents the average food consumed across eight animals. Different letters indicate statistical significance. Analysis by two-way ANOVA p_value_ ≤ 0.05 for significance. Error bars are mean ± SEM.

To explore the connection between UBE3A and PIEZO2, we generated a novel feeding assay using green fluorescent protein (GFP) expressing *Saccharomyces cerevisiae* (brewer’s yeast). Flies were starved prior to the assay and then fed GFP expressing yeast for quantification of gut distention. The GFP signal can be detected in the crop and gut of flies after consumption for accurate quantification. To determine if loss of *Dube3a* affects feeding behavior, we used a previously published *Dube3a* LOF mutant (*Dube3a*^*15b*^) [27] to compare to flies that have a known gut distention phenotype due to loss of *Piezo* [20]. Homozygous *Piezo*^*KO*^ mutants display significant gut distention, whereas *w*^*1118*^ animals feed at a basal level and do not show distention of the crop region. Quantitative comparison of abdomen sizes for *Piezo*^*KO*^ mutants to *w*^*1118*^ flies showed a significant difference in fluorescent signal and abdomen size in a region of interest (ROI) surrounding the crop (Figure 2(A)). Using *Dube3a*^*15b*^ mutants, we compared feeding behavior across *w*^*1118*^, *Piezo*^*KO*^*/Piezo*^*KO*^, *Piezo*^*KO*^*/+*, *Dube3a*^*15b*^*/+*, and trans-heterozygous *Dube3a*^*15b*^*/+;Piezo*^*KO*^*/+* animals. Quantitative fluorescent measurements revealed significantly elevated gut distention for all mutant genotypes relative to *w*^*1118*^ controls (Figure 2(B)). Notably, both *Dube3a*^*15b*^*/+* and *Piezo*^*KO*^*/+* flies exhibited comparable increases in feeding behavior. Although both the *Piezo*^*KO*^*/Piezo*^*KO*^ and trans-heterozygous groups showed slightly higher mean feeding levels than single heterozygotes, these differences did not reach statistical significance. To confirm that flies are actually eating more food as opposed to some other issue causing gut distention, we applied the widely published CApillary FEeder (CAFE) assay for food intake [28]. We found CAFE feeding behavior to parallel the GFP feeding assay, supporting the initial findings (Figure 2(C)). Collectively, these findings indicate that loss of *Dube3a* is sufficient to induce hyperphagic feeding that phenocopies those observed in *Piezo* mutants.

### *Deficiencies that uncover the* Dube3a *locus confirm* Dube3a *as a regulator of satiety signaling*

**Figure 3.**
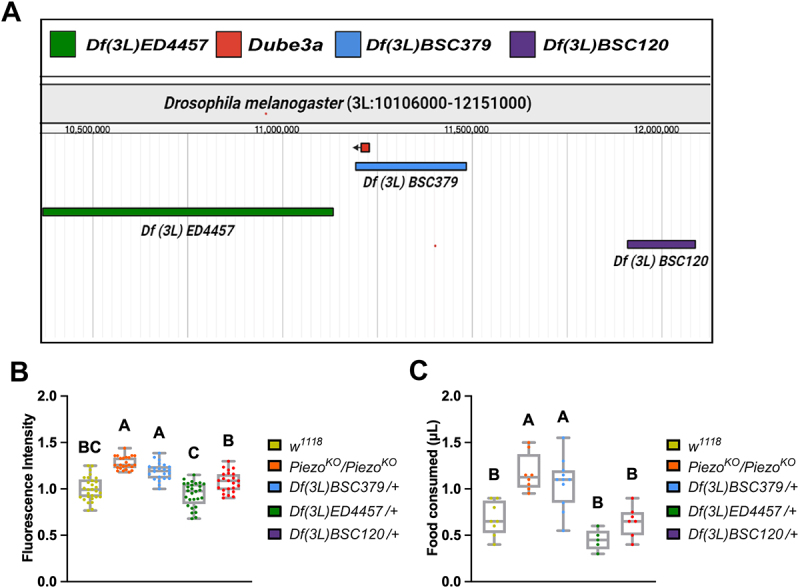
3 L deficiency flies encompassing the*Dube3a* locus recapitulate gut distention phenotype. A) genomic region encompassing *Dube3a* locus on chromosome 3 L. Genomic map captured through FlyBase JBrowse feature. B) distention analysis of *w*^*1118*^ (*n* = 29), *Piezo*^*KO*^*/Piezo*^*KO*^ (*n* = 25), *Df(3 L)BSC379* (*n* = 23), *Df(3 L)ED4457* (*n* = 28), *Df(3 L)BSC120* (*n* = 23). *Piezo*^*KO*^*/Piezo*^*KO*^ positive control and deficiency flies that uncover the *Dube3a* locus had significant gut distention phenotypes when compared to control *w*^*1118*^ flies. *Df(3 L)BSC379* had a significantly increased gut distention phenotype when compared to *Df(3 L)BSC120*. C) CAFE feeding analysis of *w*^*1118*^ (*n* = 8), *Piezo*^*KO*^*/Piezo*^*KO*^ (*n* = 8), *Df(3 L)BSC379* (*n* = 11), *Df(3 L)ED4457* (*n* = 5), *Df(3 L)BSC120* (*n* = 7). *Piezo*^*KO*^*/Piezo*^*KO*^ positive control and deficiency flies that uncover the *Dube3a* locus consumed significantly more food when compared to control *w*^*1118*^ flies. *Df(3 L)BSC379* had a significantly increased gut distention phenotype when compared to *Df(3 L)BSC120*. Each data point represents the average food consumed across eight animals. Different letters indicate statistical significance. Analysis by two-way ANOVA p_value_ ≤ 0.05 for significance. Error bars are mean ± SEM.

To confirm *Dube3a’s* role in the gut distention phenotype in a different genetic background, we tested several Bloomington Drosophila Stock Center (BDSC) deficiency (Df) lines. The Df lines have small and large deletions across the entirety of the Drosophila genome [29,30]. To ensure a direct effect of loss of *Dube3a* on satiety signaling, we performed the GFP feeding assay on three Df lines located on Drosophila 3 L. Two lines, *Df(3 L)ED4457* and *Df(3 L)BSC120* flank the *Dube3a* gene, while *Df(3 L)BSC379* is missing the *Dube3a* gene (Figure 3(A)). To eliminate other background effects, we removed *Cy*O and TM6B, *Tb, Hu* balancers by out-crossing all Df lines to *w*^*1118*^ flies prior to testing. Comparison of quantitative gut distention revealed a significant increase in feeding behavior of *Df(3 L)BSC379* but not for flanking Df lines (Figure 3(B)). While *Df(3 L)BSC120* showed an elevated fluorescent intensity signal, it was not significantly different from *w*^*1118*^. Multiple comparison analysis comparing *Df(3 L)BSC379* and *Df(3 L)BSC120* showed a significant increase in distention for *Dube3a* LOF mutants, confirming the relationship between loss of *Dube3a* and hyperphagic feeding behavior (Figure 3(B)), regardless of genetic background. We repeated this feeding experiment using the CAFE assay. Once again, the CAFE assay paralleled feeding levels to the GFP feeding assay for the Df lines (Figure 3(C)). Collectively, these Df-based analyses support the conclusion that loss of *Dube3a* is sufficient to promote hyperphagia and implicates this gene within a satiety-regulatory pathway that is functionally aligned with Piezo signaling, consistent with previous findings linking both proteins to shared neurobehavioral phenotypes [12].

### *Western blot analysis of Piezo confirms indirect regulation through* Dube3a

To further establish a connection between Dube3a and Piezo, we generated three Drosophila lines using GAL4/UAS for western blot (WB) analysis. These studies were performed using a previously identified GAL4 driver in the endogenous *Piezo* gene in flies (*Piezo*-GAL4) [31]. We crossed this GAL4 driver to UAS-*Piezo:GFP* (WT), and constructs that we generated: UAS*-Piezo:GFP;* UAS*-Dube3a:FLAG*, UAS*-Piezo:GFP*; UAS*-Dube3a-TRiP* (TRiP), and UAS*-Piezo:GFP*; UAS*-Dube3a:FLAG-C/A* (C/A). The first generated line, *UAS-Piezo:GFP; UAS-Dube3a:FLAG*, expresses the Piezo:GFP fusion protein while simultaneously overexpressing *Dube3a*-FLAG in Piezo positive cells. We generated UAS*-Piezo:GFP; Dube3a-TRiP*, that expresses the Piezo:GFP fusion protein in a *Dube3a* knockdown background via shRNA [32]. Lastly, we then generated *UAS-Piezo:GFP; UAS-Dube3a-FLAG-C/A* that overexpresses a catalytically inactive form of Dube3a with a C to A mutation at the ubiquitin ligase active site cystine residue [33] which we previously published [34].

We observed that *Piezo*>*Piezo:GFP; Dube3a:FLAG* flies failed to eclose at 25°C, suggesting lethality due to high GAL4 activity. To mitigate this, crosses were instead maintained at 18°C to reduce GAL4-driven expression [35]. Even under these conditions, only two progeny with the desired genotype successfully eclosed, indicating that overexpression of *Dube3a* in *Piezo*-expressing cells results in a nearly fully penetrant lethal phenotype. To confirm this, a Chi-square test was performed on the progeny from a cross between *Dube3a:FLAG/TM6B, Tb, Hu* flies and *Piezo-GAL4*. The analysis revealed a significant deviation from expected ratios, confirming a semi-lethal effect (χ^2^ = 14.03, df = 1, *p* = 0.0002). Due to lethality, the overexpression genotype was excluded from subsequent Western blot (WB) experiments.

**Figure 4.**
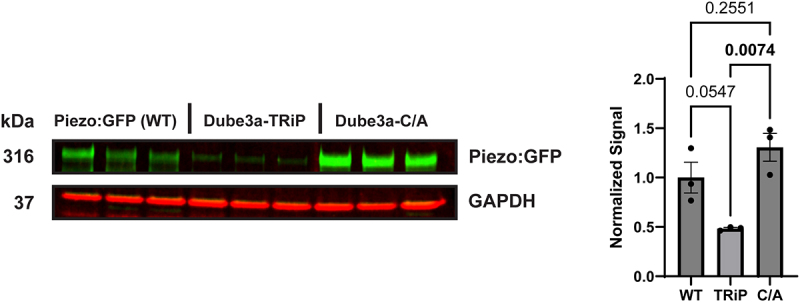
Western blot analysis of double UAS flies reveals Dube3a dependent changes in piezo protein expression. Western blot analysis of *Piezo > piezo:*GFP (WT), *Piezo > piezo:GFP; Dube3a-TRiP* (TRiP), and *Piezo > piezo:GFP; Dube3a-C/A* (C/A) animals. Knockdown of *Dube3a*, results in the significant downregulation of Piezo:GFP, with a full preservation of Piezo:GFP levels in our C/A condition. Analysis by one-way ANOVA p_value_ ≤ 0.05. Error bars are mean ± SEM. Cropped bands for Piezo:GFP and GAPDH are shown from a single membrane.

WB analysis was conducted on *Piezo > Piezo:GFP*, *Piezo > Piezo:GFP; Dube3a-TRiP*, and *Piezo > Piezo:GFP; Dube3a-FLAG-C/A* flies. These studies revealed that *Dube3a* knockdown led to a significant reduction in Piezo protein levels, while overexpression of the catalytically inactive *Dube3a-FLAG-C/A* variant preserved Piezo expression to near-endogenous levels (Figure 4). These findings reveal an indirect regulatory relationship between *Dube3a* and Piezo, providing strong support for our hypothesis that these proteins act within the same molecular pathway. Furthermore, this result reinforces prior observations suggesting a functional connection between Dube3a and Piezo signaling [12].

## Discussion

Although previously thought to be a feature unique to the pUPD class of AS [8], more recent research has revealed hyperphagia as a common symptom across all AS subtypes [10]. Collaborative work from our laboratory also recently established a connection between UBE3A and the mechanosensitive ion channel PIEZO2, leading to the hypothesis that UBE3A might indirectly regulate PIEZO channels, contributing to the both ataxia [12] and hyperphagia in AS. Here, we investigated this hypothesis and establish a link between the loss of *Dube3a* and hyperphagic feeding behavior that resembles hyperphagia in *Piezo*^*KO*^ flies, establishing that these two genes might act in the same pathway to control satiety. Biochemical investigations into Piezo protein levels in relation to *Dube3a* expression conclusively support their indirect relationship further supporting the role of Dube3a Piezo-related phenotypes.

Using publicly available single-cell RNA-sequencing datasets, we show that Dube3a and Piezo are co-expressed within enterocytes and crop-associated cell populations, identifying a physiological context in which interaction between the two pathways may occur. We developed a new assay utilizing GFP expressing yeast to quantify gut distention. Equivalent fluorescent intensities were detected between *Dube3a*^*15b*^ and *Piezo*^*KO*^ flies in this assay, suggesting a putative role for Dube3a in satiety signaling. This connection between satiety and Dube3a was confirmed using Df lines that uncover the Dube3a locus (Figure 3(B)). We previously demonstrated that *Dube3a*^*15b*^ mutants have significantly fewer actin filaments than their wild type counterparts [36]. Actin is essential for the trafficking of proteins to the membrane [37]. Studies have shown that a functional cytoskeleton is also required for proper Piezo activity [38,39]. Here, we propose that *Dube3a* loss may impair Piezo trafficking or function via dysregulation of the actin cytoskeleton. This cytoskeletal disruption could impair Piezo function and contribute to the hyperphagic feeding behavior observed in our mutants.

Hyperphagia is an underappreciated but increasingly recognized symptom across all AS subtypes. While prior work has shown that PIEZO2 function is sensitive to UBE3A levels [12], the molecular connection between these pathways remains unclear. In our study, we found that loss of *Dube3a* phenocopies *Piezo* loss in two independent feeding assays, and that both genetic and biochemical data support a functional relationship between these proteins. Although the precise intermediates remain unidentified, previous work has proposed that UBE3A may influence Piezo activity by modulating actin-regulating proteins such as cofilin [12], combined with the present results, suggest a model in which impaired cytoskeletal regulation modulates Piezo-dependent satiety signaling.

Definitive establishment of this functional relationship will require targeted rescue experiments, including re-expression of *UBE3A* in *UBE3A* LOF or knockdown backgrounds and restoration of *Piezo* expression in *UBE3A*-deficient animals to determine whether hyperphagic feeding phenotypes can be normalized. Collectively, our findings establish a mechanistic connection between UBE3A dysfunction and hyperphagic feeding behavior and present a Drosophila-based platform for further dissection of satiety regulation.

## Materials and methods

### Fly stocks

Fly stocks were maintained on standard Drosophila corn meal media (Bloomington Stock Center) and maintained at 25°C on a 12-hour light/dark cycle. The following lines were all acquired from the Bloomington Drosophila Stock Center (BDSC) *Piezo*^*KO*^ (BDSC#, 58770), *Piezo*-GAL4 (BDSC#, 78335), UAS*-Piezo:GFP* (BDSC#, 58772), *Df(3 L)BSC120* (BDSC#, 8977), *Df(3 L)ED4457* (BDSC#, 9355), and *Df(3 L)BSC379* (BDSC#, 24403). *Dube3a*^*15b*^ has been previously published as a Drosophila model for AS [27]. The *UAS-Piezo:GFP; UAS-Dube3a:FLAG, UAS-Piezo:GFP; UAS-Dube3a:FLAG-C/A*, and *UAS-Piezo:GFP; UAS-Dube3a-TRiP* lines used in this study were generated through standard genetic crosses using *UAS-Dube3a* stocks maintained in our laboratory and the *UAS-Piezo:GFP* line (BDSC# 58772).

### Gut distention assay

Adult female flies were collected post-eclosion and stored at 25°C for 3–5 d. Before testing, flies were transferred to empty vials containing only a damp kimwipe. Flies were starved for 15–18 hours at 25°C prior to feeding. GFP expressing yeast (The ODIN) were cultured at 33°C for 48 hours on G418 (gibco) yeast extract peptone dextrose (YPD) agar. GFP^+^ yeast were scrapped and layered on top of fly food in vials. Starved flies were transferred to newly created GFP^+^ yeast food vials for 1 hour. After feeding, flies were incapacitated using FlyNap (Carolina Biological Supply). Lower legs were removed and flies were placed with the abdomen facing up for imaging on a Leica fluorescent dissecting microscope. Images were collected and uploaded into FIJI (ImageJ) for analysis. The crop region of the most distended *Piezo*^*KO*^*/Piezo*^*KO*^ fly was traced, generating a normalization region of interest (ROI). The normalization ROI was then applied to all flies in the analysis to quantify distention through fluorescent intensity.

### CAFE assay

Four male and four female flies were collected post-eclosion and stored at 25°C for 3–5 d. Before testing, flies were transferred to empty vials containing only a damp kimwipe. Flies were starved for 15–18 hours at 25°C prior to feeding. Two glass capillary tubes (VWR 53,432–706) containing 7 µL of a 5% sucrose solution were administered into each vial. The starting volume was marked on each capillary tube. After 1 hour, the final volume for each tube was marked. Each fly vial had the change in volume for both capillary tubes averaged to assess amount consumed during the 1 hour testing period.

### Western blot analysis

Protein was extracted from 30 to 40 whole 3–5-day-old flies through mechanical homogenization in RIPA buffer plus Complete Protease Inhibitor Cocktail (Roche). Samples were spun down at 10,000 ×g. 30 µg of protein were loaded into each lane of 1.0 mm NuPAGE Tris-Acetate 3–8% gels (Invitrogen) and transferred to PVDF membrane (Millipore). PVDF membranes were blocked in Intercept Blocking Buffer (Li-Cor Cat# 927– 60001) for 1 hour. Membranes were incubated overnight with the following antibodies diluted in Intercept Blocking Buffer, α-GAPDH (1:5000, Abcam, ab157156), and α-GFP (1:2000 Proteintech, 50430–2-AP). The following secondary antibodies were used, IRDye 680RD Donkey α-Goat (1:5000, Li-Cor Cat# 926– 68074), and IRDye 800CW Donkey α-Rabbit (1:5000, Li-Cor Cat# 926– 32213). Samples were run ≥3 times to perform statistical analysis, and differences were considered statistically significant if *p* < 0.05. Membranes were imaged on an Azure 600 Imaging System (Azure).

## Data analysis

All data analysis was performed using Prism 10 (GraphPad). All histograms and measurements are shown as mean ± SEM. Gut distention analysis was performed using two-way ANOVA with Sidak’s multiple comparison test. Western blot analysis was performed using one-way ANOVA using Tukey’s multiple comparison. Lethality assay analysis was performed using Chi-square test. For all statistical tests, we set the α to 0.05. All figures were generated using Adobe Illustrator (Adobe).

## Data Availability

All data generated during this study are included in this manuscript or are available through Figshare (DOI:10.6084/m9.figshare.25978405). Additional information can be made available from the corresponding author upon request.
